# The aftermath of acute kidney injury: a narrative review of long-term mortality and renal function

**DOI:** 10.1186/s13054-019-2314-z

**Published:** 2019-01-24

**Authors:** Gijs Fortrie, Hilde R. H. de Geus, Michiel G. H. Betjes

**Affiliations:** 1 Department of Internal Medicine, Division of Nephrology, and Transplantation, Erasmus Medical Center, P.O. Box 2040, 3000 CA Rotterdam, The Netherlands; 2 Department of Intensive Care, Erasmus Medical Center, Rotterdam, The Netherlands

**Keywords:** Acute kidney injury, Chronic kidney disease, End-stage renal disease, Epidemiology, Survival, Comorbidity

## Abstract

Acute kidney injury (AKI) is a frequent complication of hospitalization and is associated with an increased risk of chronic kidney disease (CKD), end-stage renal disease (ESRD), and mortality. While AKI is a known risk factor for short-term adverse outcomes, more recent data suggest that the risk of mortality and renal dysfunction extends far beyond hospital discharge. However, determining whether this risk applies to all patients who experience an episode of AKI is difficult. The magnitude of this risk seems highly dependent on the presence of comorbid conditions, including cardiovascular disease, hypertension, diabetes mellitus, preexisting CKD, and renal recovery. Furthermore, these comorbidities themselves lead to structural renal damage due to multiple pathophysiological changes, including glomeruloscleroses and tubulointerstitial fibrosis, which can lead to the loss of residual capacity, glomerular hyperfiltration, and continued deterioration of renal function. AKI seems to accelerate this deterioration and increase the risk of death, CDK, and ESRD in most vulnerable patients. Therefore, we strongly advocate adequate hemodynamic monitoring and follow-up in patients susceptible to renal dysfunction. Additionally, other potential renal stressors, including nephrotoxic medications and iodine-containing contrast fluids, should be avoided. Unfortunately, therapeutic interventions are not yet available. Additional research is warranted and should focus on the prevention of AKI, identification of therapeutic targets, and provision of adequate follow-up to those who survive an episode of AKI.

## Introduction

Acute kidney injury (AKI) is defined as an abrupt loss in renal function and may be caused by a wide variety of clinical conditions. Historically, AKI was described as early as the second century AD by Claudius Galenus [1] and was initially considered a harmless transient entity with limited implications for a patient’s prognosis. However, in recent decades, this opinion has radically changed, and AKI has attracted increased interest, reflected by the exponential increase in related publications [2, 3]. Today, AKI is a frequently seen complication of hospitalization and is independently associated with a high risk of mortality and progressive deterioration of renal function, which can lead to chronic kidney disease (CKD) as well as end-stage renal disease (ESRD) and a decrease in the quality of life [4–7]. Furthermore, recent studies suggest that AKI is also a risk factor for other adverse outcomes, including stroke, cardiovascular disease, sepsis, malignancy, bone fracture, and upper gastrointestinal hemorrhage [8–16]. The results of these studies suggest that an episode of AKI plays a significant role in the patient’s long-term prognosis.

However, whether there is indeed a causal relationship between AKI and long-term adverse outcomes or whether AKI is simply an indicator of poor clinical condition remains a major topic of discussion [17–20]. A large proportion of the currently available literature consists of retrospective cohort studies that were not designed to demonstrate a causal relationship and therefore carry a substantial risk for selection bias, information bias, and residual confounding. Furthermore, the impact of AKI on long-term adverse outcomes is highly dependent on the presence of preexisting comorbidities, including cardiovascular disease, hypertension, diabetes mellitus and, in particular, preexisting CKD. Independent of AKI, most of these conditions strongly impact outcome measures such as morbidity and mortality. This narrative review offers an overview of the most relevant literature addressing the long-term impact of AKI on mortality and renal function.

## Definition and staging

For a long time, a universal definition to describe an acute deterioration in renal function was lacking. A frequently used term was acute renal failure (ARF), which was generally an umbrella term for an acute deterioration in renal function and usually used to describe a situation where emergency renal replacement therapy (RRT) was necessary. Although ARF was associated with a high hospital mortality and risk for chronic dialysis dependence [21, 22], little was known about milder episodes of renal injury, leading to a call for consensus criteria [23].

In 2004, the Acute Dialysis Quality Initiative (ADQI) group published the Risk, Injury, Failure, Loss, End-stage Renal Disease (RIFLE) criteria, which was the first consensus definition for AKI [24]. Subsequently, the RIFLE criteria were validated and, commensurate with an increased stage of severity, associated with an increased risk of short-term mortality [25, 26]. However, increasing evidence has demonstrated that even minor changes in serum creatinine are associated with an increased risk of mortality [27–29]. Therefore, in 2007, the Acute Kidney Injury Network (AKIN) published a refinement of the RIFLE criteria, and henceforth, the term ARF was officially replaced by AKI [30]. The currently used criteria, shown in Table 1, were published in 2012 by the Kidney Disease: Improving Global Outcome (KDIGO) AKI workgroup and represent a unification of the RIFLE and AKIN criteria [31].

**Table 1:** Definition of AKI by the kidney disease: improving global outcome criteria [31]

AKI stage	Serum creatinine	Urine output
I	1.5 to 2.0 times baseline within 7 days or ≥ 26.4 μmol/L within 48 h	< 0.5 ml/kg/h for 6–12 h
II	2.0 to 2.9 times baseline	< 0.5 ml/kg/h for ≥ 12 h
III	≥ 3.0 times baseline or an increase in SCr to ≥ 353.6 μmol/L or the initiation of renal replacement therapy	< 0.3 ml/kg/h for ≥ 24 h or anuria for ≥ 12 h

*AKI* acute kidney injury, *SCr* serum creatinine concentration

## AKI and long-term mortality

Even before the publication of the RIFLE criteria in 2004, multiple studies evaluated the long-term consequences of ARF and demonstrated that ARF was associated with an increased risk of mortality and other adverse outcomes. However, these conclusions were mainly based on small, retrospective, uncontrolled cohort studies performed in diverse clinical settings. With the lack of consensus criteria for ARF, this variation resulted in significant differences in study outcomes, which made it difficult to generalize these results to other populations and use them in clinical practice.

One of the first studies that described the long-term effect of AKI compared to the outcomes of patients without AKI after thoracic surgery (*n* = 88) was performed in 1994 by Schepens et al. [32]. During the postsurgical period, 14% of the cohort developed AKI requiring RRT. The 5-year survival rate was 20% for these patients but was 62% for the patients without RRT (*P* = 0.001). This paper triggered the publication of numerous papers on the association between AKI and long-term mortality, which further led to an increase in the quality and sample size of these studies. In 2009, *Coca* et al. performed a systematic review and meta-analysis of 48 studies with follow-up times of between 6 months and 17 years [4]. The clinical setting of the incorporated studies was heterogeneous and included patients undergoing cardiac surgery, percutaneous coronary intervention, and liver or lung transplantation, as well as general ICU patients. Fifteen studies were eligible for long-term survival analysis and provided data on long-term mortality in AKI patients (*n* = 8350) as well as in non-AKI controls (*n* = 90,753). Overall, the mortality rate was significantly different between the AKI patients who survived hospital admission (mortality rate = 8.9 per 100 person-years) and the non-AKI controls (4.3 per 100 person-years). Furthermore, the risk of death increased proportionally with the severity of AKI. Due to the heterogeneous AKI definitions used in the studies, the patients were stratified into three groups: mild, moderate, and severe AKI. Mild AKI was defined as an increase in serum creatinine of > 25% or a decrease in creatinine clearance of > 10%; moderate AKI was defined as an increase in serum creatinine of > 50%, 100%, or > 1.0 mg/dl or a creatinine concentration of > 1.7 mg/dl; and severe AKI was defined as a necessity for RRT. The pooled rate ratios for mild, moderate, and severe AKI compared to that of the non-AKI controls were 1.67, 2.70, and 3.09, respectively.

While the analyses by Coca et al. included only studies with a relatively small study population, the results of more recently published studies with large sample sizes are presented in Table 2A [33–42]. The largest study, by Lafrance et al., demonstrated in a retrospective analysis among US veterans (*n* = 864,933) that patients with an episode of AKI not requiring RRT had an adjusted hazard ratio (HR) of 1.41 for long-term mortality (95% CI = 1.39–1.43) [38]. When stratified by AKI severity according to the AKIN definition, the adjusted HRs were 1.36, 1.46, and 1.59 for stages I, II, and III (without RRT), respectively (*P* < 0.001 for the trend). Similar results were shown for subgroup analyses restricted to patients who survived at least 3 or 6 months after discharge; even more interestingly, the negative effect of AKI persisted in patients who showed only short-term impairment in renal function during hospitalization. These results demonstrate that even a short transient deterioration in renal function is associated with a poorer outcome.

**Table 2:** Summary of the largest original investigations on long-term risk of mortality or ESRD in adult patients who experienced AKI

Author	Setting	Population	Number	Follow-up	AKI definition	Adjusted risk		Comments
A. Long-term risk of mortality								
Bihorac et al. [33]	ICU (surgical)	Hospital survivors	10,518	Max 14 years	RIFLE criteria	R I F	HR (95% CI) = 1.18 (1.08–1.29) HR (95% CI) = 1.43 (1.29–1.59) HR (95% CI) = 1.57 (1.40–1.75)	–
Coca et al. [34]	Noncardiac surgery	Hospital survivors	35,302	Mean 3.7 years	AKIN criteria	I II III	HR (95% CI) = 1.24 (1.17–1.31) HR (95% CI) = 1.64 (1.43–1.88) HR (95% CI) = 1.96 (1.63–2.37)	*Only diabetic veterans included.*
Fuchs et al. [35]	ICU (overall)	60-day survivors	12,399	Max 2.0 years	AKIN criteria	I II III	HR (95% CI) = 1.26 (1.14–1.40) HR (95% CI) = 1.28 (1.11–1.47) HR (95% CI) = 1.61 (1.30–1.99)	–
Ishani et al. [36]	Overall hospitalization	Hospital survivors	233,803	Max 2.3 years	ICD-9 code	AKI	HR (95% CI) = 2.38 (2.31–2.46)	*Only elderly patients ≥ 67 years of age included.*
James et al. [37]	Coronary angiography	All patients	14,782	Median 1.6 years	AKIN criteria	I II/III	HR (95% CI) = 2.00 (1.69–2.36) HR (95% CI) = 3.72 (2.92–4.76)	–
Lafrance et al. [38]	Overall hospitalization	90-day survivors	864,933	Mean 2.3 years	AKIN criteria	I II III	HR (95% CI) = 1.36 (1.34–1.38) HR (95% CI) = 1.46 (1.42–1.50) HR (95% CI) = 1.59 (1.54–1.65)	*Only veterans included. AKI requiring RRT excluded.*
Liotta et al. [39]	CABG	All patients	25,665	Mean 6.0 years	Mild ΔSCr 0.0–0.3 mg/dl Moderate ΔSCr 0.3–0.5 mg/dl Severe ΔSCr ≥ 5.0 md/dl	Mild Moderate Severe	HR (95% CI) = 1.07 (1.00–1.15) HR (95% CI) = 1.33 (1.19–1.48) HR (95% CI) = 2.11 (1.92–2.32)	–
Parikh et al. [40]	AMI	Hospital survivors	147,007	Max 10.0 years	Mild ΔSCr 0.3–0.4 mg/dl Moderate ΔSCr 0.5–0.9 mg/dl Severe ΔSCr ≥ 1.0 md/dl	Mild Moderate Severe	HR (95% CI) = 1.15 (1.12–1.18) HR (95% CI) = 1.23 (1.20–1.26) HR (95% CI) = 1.33 (1.28–1.38)	*Only elderly patients ≥ 65 years of age included.*
Rimes-Stigare et al. [41]	ICU (overall)	All patients	103,363	Median 2.1 years	Temporary RRT or ICD-10 code or ARF reported in APACHE score or serum creatinine > 354 μmol/L	AKI	MMR (95% CI) = 1.15 (1.09–1.21)	–
Ryden et al. [42]	CABG	All patients	27,929	Mean 5.0 years	Mild ΔSCr 0.3–0.4 mg/dl Moderate ΔSCr 0.5–0.9 mg/dl Severe ΔSCr ≥ 1.0 md/dl	Mild Moderate Severe	HR (95% CI) = 1.30 (1.17–1.44) HR (95% CI) = 1.65 (1.48–1.83) HR (95% CI) = 2.68 (2.37–3.03)	–
B. Long-term risk of ESRD								
Ishani et al. [36]	Overall hospitalization	Hospital survivors	233,803	Max 2.3 years	ICD-9 code	AKI	HR (95% CI) = 6.74 (5.90–7.71)	*Only elderly patients ≥ 67 years of age included.*
James et al. [37]	Coronary angiography	All patients	14,782	Median 1.6 years	AKIN criteria	I II/III	HR (95% CI) = 4.15 (2.32–7.42) HR (95% CI) = 11.74 (6.38–21.59)	–
Rimes-Stigare et al. [41]	ICU (overall)	All patients	103,363	Median 2.1 years	Temporary RRT or ICD-10 code or ARF reported in APACHE score or serum creatinine > 354 μmol/L	AKI	IRR (95% CI) = 24.1 (13.9–42.0)	–
Ryden et al. [61]	CABG	All patients	29,330	Mean 4.3 years	AKIN criteria	I II/III	HR (95% CI) = 2.92 (1.87–4.55) HR (95% CI) = 3.81 (2.14–6.79)	–

This table includes only studies with > 10,000 patients. Studies that only evaluated the impact of AKI requiring RRT are not included

*AKI* acute kidney injury, *AKIN* Acute Kidney Injury Network, *AMI* acute myocardial infarction, *APACHE* Acute Physiology And Chronic Health Evaluation, *ARF* acute renal failure, *CABG* coronary artery bypass grafting, *CI* confidence interval, *HR* hazard ratio, *ICD* International Classification of Diseases, *ICU* intensive care unit, *IRR* incidence rate ratio, *MMR* mortality rate ratio, *RIFLE* Risk, Injury, Failure, Loss, End-stage Renal Disease, *RRT* renal replacement therapy, *SCr* serum creatinine concentration

In addition to the severity of AKI, the risk of long-term mortality is strongly determined by other clinical and demographic patient characteristics, including age [43], baseline renal function [43, 44], malignancy [43], severe sepsis and septic shock [45, 46], recurrent episodes of AKI [47], and particularly, renal recovery [33, 34, 38, 48–58]. There is a gradual association between the proportion of early post-AKI renal recovery and the long-term mortality risk. As shown in Table 3, the risk of death increases significantly in patients with partial or no renal recovery following AKI. In addition, the vast majority of patients who experienced an episode of AKI have one or more comorbid conditions, which, given the strong relationship between preexisting comorbidities and the impact of AKI, may result in the overestimation of long-term mortality risk in patients with a low comorbidity burden. In 2015, Fortrie et al. performed a retrospective cohort study on the long-term sequelae of AKI requiring RRT in critically ill patients without any comorbid conditions. This study demonstrated that in-hospital mortality was equally high among those with or without any comorbid conditions. However, the study also demonstrated that patients without comorbidity that survived an episode of AKI and were discharged from the hospital had a good long-term prognosis; furthermore, compared to survival in the average Dutch population, no increased risk for mortality was found [20]. These conclusions are limited by the retrospective nature and relatively small sample size of the study, as only 96 of the 1067 patients were not known to have any comorbidities. Nevertheless, the results of this study are intriguing because they add evidence supporting the concept that comorbidity is a key player in the long-term impact of AKI.

**Table 3:** Summary of investigations evaluating the impact of post-AKI renal recovery on mortality and/or CKD and ESRD compared to no-AKI controls

Author	Setting	Number	Follow-up	AKI definition	Renal recovery definition		Mortality risk	CKD/ESRD risk*
Bihorac et al. [33]	ICU (surgical)	10,518	Max 14.0 years	RIFLE criteria	Complete Partial Nonrecovery	ΔSCr at discharge ≤ 50% ΔSCr at discharge > 50% RRT at discharge	HR (95% CI) = 1.20 (1.10–1.31) HR (95% CI) = 1.45 (1.32–1.58) HR (95% CI) = 2.76 (2.09–3.43)	–
Brown et al. [48]	Cardiac surgery	4873	Mean 2.5 years	AKIN criteria	Transient Nonrecovery	ΔSCr at 1–2 days ≥ 50% or > 0.3 mg/dl ΔSCr at 3–6 days ≥ 50% or > 0.3 mg/dl ΔSCr at ≥ 7 days ≥ 50% or > 0.3 mg/dl ΔSCr at discharge ≥ 50%	HR (95% CI) = 1.51 (1.19–1.91) HR (95% CI) = 1.74 (1.34–2.26) HR (95% CI) = 3.45 (2.75–4.34) HR (95% CI) = 5.75 (4.10–8.07)	–
Bucaloiu et al. [49]	Overall hospitalization	20,028	Mean 3.3 years	AKIN criteria	Recovery	ΔeGFR at day 90 ≤ 10%	HR (95% CI) = 1.48 (1.19–1.82)	HR (95% CI) = 1.91 (1.75–2.09)
Coca et al. [34]	Noncardiac surgery	35,302	Mean 3.7 years	AKIN criteria	Transient	ΔSCr at 1–2 days ≥ 50% or > 0.3 mg/dl ΔSCr at 3–6 days ≥ 50% or > 0.3 mg/dl ΔSCr at ≥ 7 days ≥ 50% or > 0.3 mg/dl	HR (95% CI) = 1.15 (1.07–1.23) HR (95% CI) = 1.50 (1.36–1.66) HR (95% CI) = 2.01 (1.77–2.28)	–
Han et al. [50]	CABG	1899	Median: 5.0 years	KDIGO criteria	Recovery Nonrecovery	SCr at 3 months ≤ baseline SCr SCr at 3 months > baseline SCr	HR (95% CI) = 1.68 (1.35–2.10) HR (95% CI) = 2.06 (1.52–2.79)	–
Hobson et al. [51]	Cardiothoracic surgery	2973	Max 10.0 years	RIFLE criteria	Complete Partial Nonrecovery	ΔSCr at discharge ≤ 50% ΔSCr at discharge > 50% RRT at discharge	HR (95% CI) = 1.28 (1.11–1.48) HR (95% CI) = 1.49 (1.27–1.74) HR (95% CI) = 3.79 (2.46–5.74)	–
Jones et al. [52]	Overall hospitalization	3809	Median 2.5 years	AKIN criteria	Recovery	ΔSCr at day 7 < 10%	HR (95% CI) = 1.08 (0.93–1.27)	HR (95% CI) = 3.82 (2.81–5.19)
Kuijk et al. [68]	Major vascular surgery	1308	Median 5.0 years	ΔSCr > 10% vs. baseline	Recovery Nonrecovery	ΔSCr at day 3 ≤ 10% ΔSCr at day 3 > 10%	–	RR (95% CI) = 3.40 (2.70–4.10) RR (95% CI) = 3.60 (2.80–4.40)
Lafrance et al. [38]	Overall hospitalization	864,933	Mean 2.3 years	AKIN criteria	Recovery	ΔeGFR at discharge ≤ 10%	HR (95% CI) = 1.47 (1.43–1.51)	–
Loef et al. [53]	Cardiac surgery	843	Max 14.3 years	ΔSCr ≥ 25% vs. baseline	Recovery Nonrecovery	SCr at discharge ≤ baseline SCr SCr at discharge > baseline SCr	HR (95% CI) = 1.66 (1.09–2.53) HR (95% CI) = 1.72 (1.00–2.96)	–
Maioli et al. [54]	Coronary angiography	1490	Median 3.8 years	ΔSCr > 0.5 mg/dl vs. baseline	Recovery Nonrecovery	ΔSCr at 3 months < 25% ΔSCr at 3 months ≥ 25%	HR (95% CI) = 1.30 (1.10–1.70) HR (95% CI) = 2.30 (1.30–4.00)	–
Mehta et al. [55]	CABG	10,415	Median: 7.0 years	ΔSCr ≥ 50% or ≥ 0.7 mg/dl vs. baseline	Complete Partial Nonrecovery	SCr at day 7 ≤ baseline SCr ΔSCr at day 7 < 50% or < 0.7 mg/dl ΔSCr at day 7 ≥ 50% or ≥ 0.7 mg/dl	HR (95% CI) = 1.21 (1.07–1.37) HR (95% CI) = 1.58 (1.36–1.82) HR (95% CI) = 1.42 (1.27–1.59)	–
Pannu et al. [56]	Overall hospitalization	190,714	Mean 2.8 years	ΔSCr ≥ 100% vs. baseline or RRT requirement	No AKI Recovery Nonrecovery	No AKI criteria ΔSCr at 90 days ≤ 25% ΔSCr at 90 days > 25%	HR (95%CI) = 0.69 (0.64–0.75) Reference HR (95% CI) = 1.28 (1.13–1.46)	HR (95% CI) = 0.63 (0.54–0.74) Reference HR (95% CI) = 5.59 (3.77–5.58)
Wu et al. [57]	ICU (surgical)	9425	Median 4.8 years	RIFLE criteria	AKI (CKD-) recovery AKI (CKD-) nonrecovery AKI (CKD+) recovery AKI (CKD+) nonrecovery	ΔSCr at discharge < 50% ΔSCr at discharge > 50% ΔSCr at discharge < 50% ΔSCr at discharge > 50%	HR (95% CI) = 1.96 (1.78–2.16) HR (95% CI) = 2.18 (1.24–3.84) HR (95% CI) = 3.00 (2.35–3.84) HR (95% CI) = 4.59 (3.20–6.45)	HR (95% CI) = 4.50 (2.43–8.35) HR (95% CI) = 60.95 (24.13–153.97) HR (95% CI) = 74.07 (38.82–141.32) HR (95% CI) = 212.73 (105.53–428.83)
Xu et al. [58]	Cardiac surgery	3245	Max 2.0 years	KDIGO criteria	Recovery Nonrecovery	ΔSCr at discharge ≤ 44 μmol/L ΔSCr at discharge > 44 μmol/L	RR (95% CI) = 1.79 (1.20–2.49) RR (95% CI) = 8.64 (6.04–12.34)	RR (95% CI) = 1.92 (1.37–2.69) RR (95% CI) = 15.05 (10.88–20.82)

*AKI* acute kidney injury, *AKIN* Acute Kidney Injury Network, *AMI* acute myocardial infarction, *CABG* coronary artery bypass grafting, *CI* confidence interval, *CKD* chronic kidney disease, *ESRD* end-stage renal disease, *HR* hazard ratio, *ICU* intensive care unit, *KDIGO* Kidney Disease: Improving Global Outcome, *RIFLE* Risk, Injury, Failure, Loss, End-stage Renal Disease, *RR* relative risk, *RRT* renal replacement therapy, *SCr* serum creatinine concentration

*The chosen endpoints differed between the individual studies. Bucaloiu et al.: new CKD (eGFR < 60 ml/min), Jones et al.: new CKD (eGFR < 60 ml/min), Kuijk et al.: new CKD (eGFR < 60 ml/min and eGFR decrease ≥ 25% compared to baseline), Pannu et al.: need for chronic RRT dependence or doubling of the SCr compared to baseline, Wu et al.: chronic RRT dependence, Xu et al.: eGFR < 30 ml/min

## AKI and long-term risk for CKD and ESRD

While the association between AKI and long-term mortality seems to be based on a complex interplay between AKI and many other patient-specific factors, this interplay is even more complex for the association between AKI and long-term deterioration in renal function. Many recent studies have described the association between AKI and progression to CKD or even ESRD, which has led to a discussion on whether there is a causal relationship between AKI and CKD or whether this association is simply the result of methodological differences and preexisting comorbidities such as diabetes, hypertension, cardiovascular disease, and of course, preexisting CKD [17, 18, 59, 60]. In 2012, Coca et al. demonstrated, in another meta-analysis including 13 studies with a maximum follow-up of 75 months, a strong association between AKI and the development of CKD as well as ESRD, with adjusted HRs of 8.82 (95% CI = 3.05–25.48) and 3.10 (95% CI = 1.91–5.03), respectively [5]. Furthermore, those authors demonstrated that the risk of CKD as well as that of ESRD increased in a graded fashion with AKI severity. These results are in accordance with the results of the large population-based studies that evaluated the risk of ESRD in AKI survivors presented in Table 2B [36, 37, 41, 61]. In addition, a large study by Lo et al. that included more than 500,000 patients with a baseline estimated glomerular filtration rate (eGFR) of > 45 ml/min/1.73 m^2^ demonstrated that AKI requiring RRT was strongly associated with the development of stage 4 or 5 CKD, with an adjusted HR of 28.1 (95% CI = 21.1–37.6) [62].

However, the AKI survivor population is very heterogeneous, and AKI etiology varies widely. Therefore, identifying individuals with the highest risk of renal deterioration is greatly important. In addition to AKI, other factors associated with an increased risk of CKD or ESRD include higher age [43, 49, 56], lower baseline renal function [36, 43, 44, 49, 57, 63, 64], diabetes [36, 56], hypertension [36, 49, 63, 64], chronic heart failure [49, 56], low serum albumin [49], proteinuria [64], liver failure [63], higher Charlson comorbidity index score [49, 63], and recurrent episodes of AKI [64]. In summary, those with the highest risk of progression towards CKD or ESRD after an episode of AKI are those who already have an increased risk for CKD progression independent of an episode of AKI. Additionally, the complexity of this association is increased even more because the vast majority of the aforementioned risk factors are associated with an increased risk of AKI itself [49, 65–67].

In 2009, Ishani et al. demonstrated in 200,000 hospitalized elderly that patients with AKI but without preexisting CKD as well as patients with preexisting CKD but without AKI have an increased risk of developing ESRD. In addition, those authors demonstrated that an episode of AKI in patients with CKD exponentially potentiates the development of ESRD (adjusted HR = 41.2, 95% CI = 34.6–49.1) [36]. These results are in accordance with those published by Wu et al. in 2010 [57], which demonstrated in a population of over 9000 surgical ICU patients with a median follow-up of 4.6 years that patients with both AKI and CKD had an adjusted HR of 91.6 (95% CI = 49.3–170.1) for ESRD. Furthermore, a subgroup analysis was performed in a cohort stratified by renal recovery at hospital discharge, which was defined as a serum creatinine concentration at discharge of < 50% above the baseline serum creatine concentration. Patients who experienced an episode of acute-on-chronic kidney disease without renal function recovery at hospital discharge had the greatest risk for ESRD compared to patients without AKI and CKD (adjusted HR = 212.7), followed by those with acute-on-chronic kidney disease with recovery (HR = 74.1), those with AKI without recovery (HR = 61.0), those with CKD without AKI (HR = 42.6), and those with AKI with recovery (HR = 4.5) (all *P* values < 0.001). Although no consensus criteria for renal recovery have been developed, these results are in accordance with the results of most other recently published studies that evaluated the impact of renal recovery or post-AKI renal function on CKD or ESRD (Table 3) [49, 52, 56–58, 68]. In contrast, one postoperative study by van Kuijk et al. did not demonstrate a gradual relationship between AKI with or without renal recovery and CKD, and the relative risk was equally high in both groups [68]. This difference could result from the short timeframe in which renal recovery was determined (day 3 after diagnosis). Furthermore, the highest incidence rate of complications after AKI is observed during the first consecutive year but appears to decline in subsequent years. Fortrie et al. showed a strong association between AKI and impaired renal function 1 year following transplantation in a cohort of patients who underwent cardiac transplantation [69]. However, with longer follow-up, only AKI requiring RRT was associated with further deterioration of renal function. In contrast to AKI, renal function at 1 year following transplantation was strongly associated with further renal deterioration [70].

**Fig. 1:**
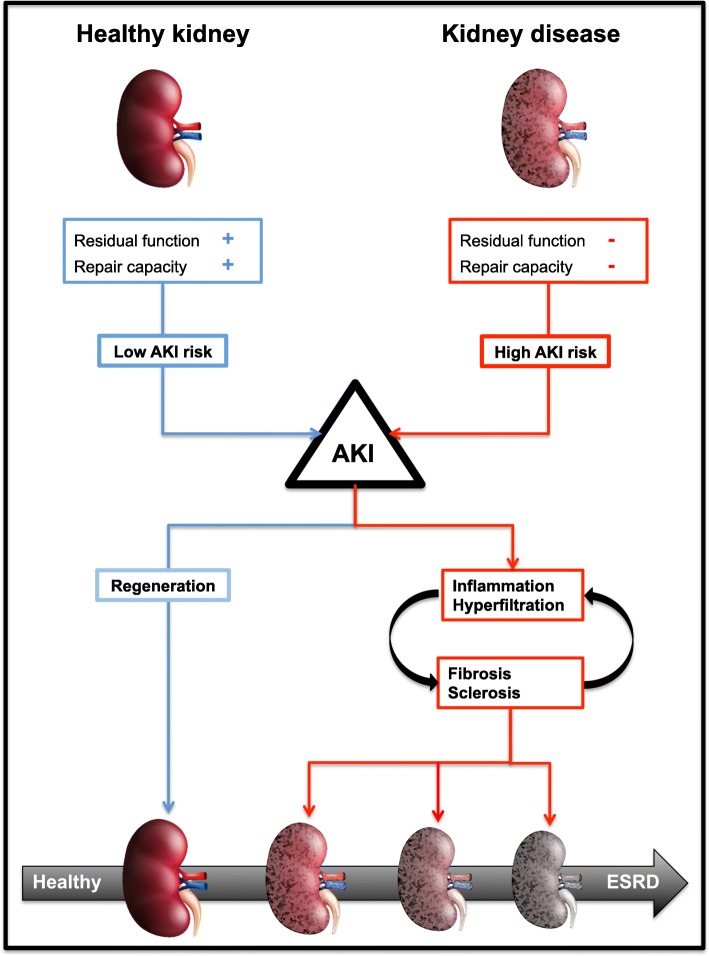
A schematic representation of the long-term sequelae of AKI. The kidney figures represent the baseline renal function. AKI, acute kidney injury; ESRD, end-stage renal disease

In conclusion, AKI is statistically an independent risk factor for CKD as well as for ESRD. However, the magnitude of this risk depends on the presence of premorbid conditions and the susceptibility to accelerated injury with impaired renal recovery. In other words, the impact of AKI on long-term outcomes depends on the residual renal function and repair capacity after renal stress. Furthermore, hyperfiltration can camouflage structural renal damage in a previously healthy kidney because the estimated glomerular filtration rate can be preserved for an extended duration. However, eventually, the renal self-repair capacity is exceeded due to continued degenerative processes, and the impact of AKI accelerates progression to CKD and ESRD. A schematic representation of this concept is shown in Fig. 1.

## Acute and long-term pathophysiological changes associated with AKI

The results of epidemiological clinical research are in line with the suggested pathophysiological mechanisms underlying a poor renal outcome after AKI. Currently, the pathophysiology of AKI is still incompletely understood and is mediated by a complex interplay among multiple pathophysiological processes. Whether this process eventually results in continued irreversible renal damage is highly dependent on residual renal function and repair capacity. Over the past decade, more insight has become available on pathophysiologic mechanisms acting during AKI. While these insights are primarily based on animal studies, they provide knowledge on the complex interplay of factors leading to kidney injury and offer potential targets for future therapy [71]. Because the etiology of AKI is very heterogeneous, AKI can initiate multiple pathophysiological pathways, often resulting from an imbalance in oxygen supply and demand. This imbalance results in hypoxemia and oxidative stress, which subsequently lead to endothelial damage, immune system activation and inflammation, and interstitial edema and vasoconstriction, which in return further decrease the oxygen supply [72]. Furthermore, dependent on the etiology of AKI, other factors may contribute to the development of AKI, including venous renal congestion due to heart failure, altered microcirculatory flow distribution due to sepsis, microthrombi due to vascular occlusive disease, tubular obstructions due to cast nephropathy, immune complex precipitation, or postrenal obstruction [73–76].

In minor and transient episodes of kidney injury, the kidney possesses multiple mechanisms to limit this damage and even the possibility of tissue repair [71, 77]. However, in prolonged and severe episodes of kidney injury, these mechanisms fail. In patients with sustained AKI or preexisting CKD, the integrity and connection between the peritubular capillaries and the tubular cells are lost, resulting in tubular dedifferentiation, apoptosis, continued capillary damage, and chronic hypoxemia. These events subsequently activate multiple proinflammatory, profibrotic pathways, which further impairs renal integrity, and the tubular regeneration capacity [78–81]. Ultimately, this cascade will result in a self-sustaining process of persistent inflammation, hyperfiltration, progressive tubular damage, glomerulosclerosis, and tubulointerstitial fibrosis that eventually leads to CKD, ESRD, and associated complications [81–83]. However, this process is also the cornerstone in the development of CKD in general. Therefore, determining whether the continued renal deterioration is the result of AKI as an independent entity or simply an indicator of progressive CKD is difficult. However, these results indicate that AKI, at a minimum, accelerates these processes (Fig. 1).

## Implications for the bedside and a glimpse into the future

Unfortunately, the increased knowledge and awareness of AKI still has a limited impact on clinical practice. In summary, the current treatment regime for AKI has not changed in recent decades and stresses preventive measures, such as limiting nephrotoxic medication and iodine-containing contrast fluids and providing adequate fluid expansion during the use of predictable potential stressors [84, 85]. Additional experimental interventions, including remote ischemic preconditioning and pharmacological interventions, have been studied but have limited effects [86]. The results of the long-awaited *STOP-AKI trial* are recently published [87]. This multicenter double-blind placebo-controlled clinical trial evaluates the safety and efficacy of human recombinant alkaline phosphatase as an anti-inflammatory treatment for patients with septic AKI. While the first published results were promising, human recombinant alkaline phosphatase did not improve short-term renal function. However, the authors demonstrated that there was a significant difference in mortality and major adverse kidney events in favor of the patients treated with recombinant alkaline phosphatase. Therefore, additional research is warranted to evaluate the role of recombinant alkaline phosphatase in the treatment of AKI.

Those at risk for AKI require consequent hemodynamic monitoring, including adequate follow-up of urine output, which is mandatory for the early detection of AKI. Therefore, automated electronic alerts (E-Alerts) for AKI could facilitate the early recognition of AKI. While it seems logical that such an intervention would raise awareness and improve patient care, the results of studies on this topic are conflicting [88–91]. For example, Wilson et al. recently performed a large randomized clinical trial including approximately 2400 patients and demonstrated that the use of E-Alerts had no beneficial effect [91]. The use of E-Alerts may even be potentially harmful and can lead to overtreatment when the data are misinterpreted.

However, it is of pivotal importance that AKI survivors preserve renal function as much as possible to prevent the further acceleration of renal deterioration. Therefore, tight control of hypertension, proteinuria, diabetes mellitus, cardiovascular disease, and other relevant comorbidities seems warranted, as the clinical efficacy of these strategies has been proven to slow or prevent the progression of CKD [92, 93]. In contrast to patients with known CKD, only a small proportion of patients who experience an episode of AKI, even an episode requiring RRT, are offered follow-up by a nephrologist. In 2012, Siew et al. demonstrated in approximately 4000 AKI survivors that the cumulative incidence of referral to a nephrologist in the first year was only 8.5%, while the mortality rate during this surveillance period was 22%. Furthermore, the severity of AKI did not affect the referral rate [94]. Subsequently, Harel et al. studied the association between follow-up by a nephrologist within 90 days post-AKI and survival. Those authors used propensity score analyses to match patients with and without follow-up by a nephrologist and reported that, overall, only 41% of the patients had follow-up in the outpatient clinic and that these patients were most likely those with preliminary CKD [95]. More interestingly, Harel et al. found that post-AKI outpatient follow-up was associated with a 24% reduction in mortality after a surveillance period of 2 years. While these results potentially provide a solution to reduce the long-term complications of AKI, clinical trials are required for improved clarity. Currently, a large randomized clinical trial is underway in Canada to address this issue [96]. Publication of the results is expected in 2022 and may have important implications for the long-term follow-up, treatment and outcome of AKI survivors.

## Conclusions

AKI is a highly complex syndrome associated with increased mortality and loss of renal function in the long term. Although most evidence has been obtained through retrospective research, the results of the numerous well-designed large studies indicate that a causal relationship between AKI and a worsened long-term prognosis is highly likely. Furthermore, these studies have offered essential insight into the populations with the greatest risk for poor prognosis, including the elderly, those with preexisting comorbidities, and particularly, those with preexisting renal impairment. While these findings are undoubtedly of great importance, they still have limited significance for clinical practice, as effective therapeutic interventions are not yet available. Therefore, the main focus of future research should be on the prevention of AKI, the identification of therapeutic targets, and the provision of adequate follow-up and treatment to preserve the renal function of patients who survive an episode of AKI.

## Abbreviations

ADQI: Acute dialysis quality initiative
AKI: Acute kidney injury
AKIN: Acute Kidney Injury Network
AMI: Acute myocardial infarction
ARF: Acute renal failure
CABG: Coronary artery bypass grafting
CKD: Chronic kidney disease
eGFR: Estimated glomerular filtration rate
ESRD: End-stage renal disease
ICU: Intensive care unit
KDIGO: Kidney Disease: Improving Global Outcome
MDRD: Modification of diet in renal disease
RIFLE: Risk, Injury, Failure, Loss, End-stage Renal Disease
